# Supplementary material for: Mitochondrial mechanics nucleates axonal jamming and swelling
Source: ArXiv. 2026 Apr 23:arXiv:2604.22024v1. Preprint. [Version 1] (PMC13131845)
Supplement: 1 [file NIHPP2604.22024V1-supplement-1.pdf]

10 Supplementary Material

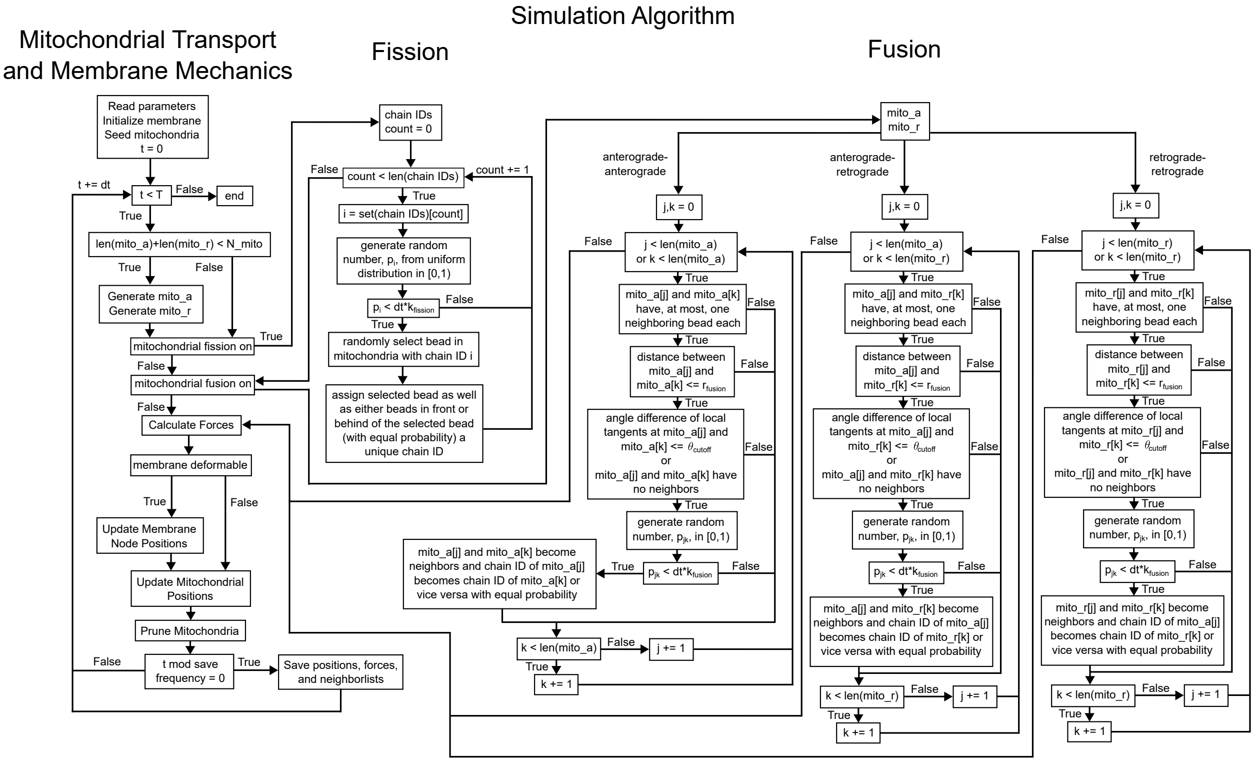

Figure S1: Graphical Algorithm of Mitochondrial transport in axon - including mitochondrial transport and membrane mechanics (left), mitochondrial fission (middle), and mitochondrial fusion (right).

Movie 1: Low Density ($\phi = 0.3\phi_{\max}$), low stiffness ($k_{\mathrm{b,mito}} = 10^{-21}$ Nm^2), $N_{\mathrm{chain}} = 5$ characteristic simulation. Jamming is minimal and anterograde and retrograde populations can pass by one another easily.
Movie 2: High Density ($\phi = 0.9\phi_{\max}$), low stiffness ($k_{\mathrm{b,mito}} = 10^{-21}$ Nm^2), $N_{\mathrm{chain}} = 5$ characteristic simulation. Jamming is prominent and anterograde and retrograde populations require extended time to pass by one another.
Movie 3: High Density ($\phi = 0.9\phi_{\max}$), high stiffness ($k_{\mathrm{b,mito}} = 10^{-19}$ Nm^2), $N_{\mathrm{chain}} = 5$ characteristic simulation. Jamming is prevalent but the persistent anistropy of the mitochondrial chains aid in alleviating the jam.
Movie 4: High Density ($\phi = 0.9\phi_{\max}$) $N_{\mathrm{chain}} = 1$ characteristic simulation. Jamming is prominent and the granularity of the mitochondria reinforce the jamming severity.
Movie 5: High Density ($\phi = 0.9\phi_{\max}$), high fission rate ($k_{\mathrm{fission}} = 100$ s^{-1}), $N_{\mathrm{chain}} = 5$ characteristic simulation. Jamming is prominent and plentiful fission events resulting in granular mitochondria reinforce the jamming severity.
Movie 6: High Density ($\phi = 0.9\phi_{\max}$), high fusion rate ($k_{\mathrm{fusion}} = 100$ s^{-1}), $N_{\mathrm{chain}} = 5$ characteristic simulation. Jamming is minimized as plentiful fusion events give rise to highly anisotropic tubular mitochondria that can easily pass by one another.
Movie 7: High Density ($\phi = 0.9\phi_{\max}$), $N_{\mathrm{chain}} = 5$, with membrane deformations characteristic simulation. Elongated mitochondrial morphologies exert less stresses on the membrane than fragmented spherical mitochondria.

Table S1: Figure Parameter Variations

Figure	Varied Parameter(s)	Values
1	-	-
2	Density (ϕ) [1]	$0.3\phi_{\text{max}}, 0.6\phi_{\text{max}}, 0.9\phi_{\text{max}}$
3	Mitochondrial bending rigidity ($k_{\text{b,mito}}$) [Nm^2]<br>Density (ϕ) [1]	$10^{-21}, 10^{-20}, 10^{-19}$<br>$0.3\phi_{\text{max}}, 0.6\phi_{\text{max}}, 0.9\phi_{\text{max}}$
4	Number of beads per mitochondria (N_{chain}) [#]<br>Density (ϕ) [1]	1, 2, 3, 4, 5<br>$0.3\phi_{\text{max}}, 0.6\phi_{\text{max}}, 0.9\phi_{\text{max}}$
5	Fusion rate (k_{fusion}) [s^{-1}]<br>Fission rate (k_{fission}) [s^{-1}]	0, 0.01, 0.1, 1.0, 10, 100<br>0, 0.01, 0.1, 1.0, 10, 100
6	Fission rate (k_{fission}) [s^{-1}]<br>Number of beads per mitochondria (N_{chain}) [#]	0, 0.1, 1, 10<br>1, 5

Movie 8: High Density ($\phi = 0.9\phi_{\text{max}}$), $N_{\text{chain}} = 1$, with membrane deformations characteristic simulation. Membrane dilation at the collision site locally increase the cross sectional area, providing more paths for mitochondria to escape the jamming.

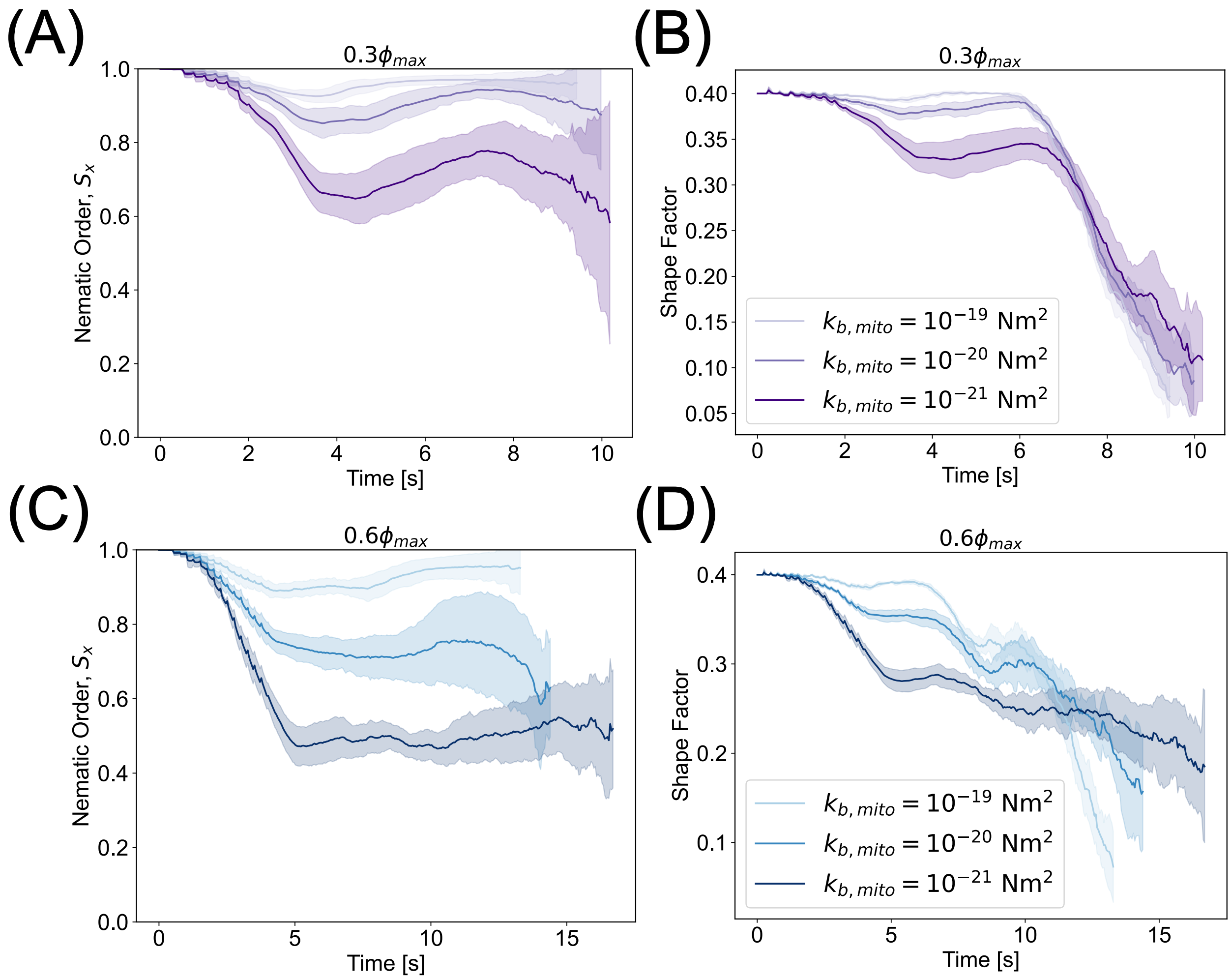

Figure S2: **Nematic and shape factor analysis of intermediate and sparse densities for varying bending stiffness.** (A,B) Nematic order and shape factor of lowest densities, $\phi = 0.3\phi_{\text{max}}$, shows 10^{-19} Nm^2 and 10^{-20} Nm^2 bending rigidities recover initial morphologies, while 10^{-21} Nm^2 only partially recovers. (C-D) Intermediate densities exhibit very similar behavior to their high density counterparts described in the main text. Curves and shaded regions represent the mean and standard deviation, respectively, of ten simulations per parameter set.

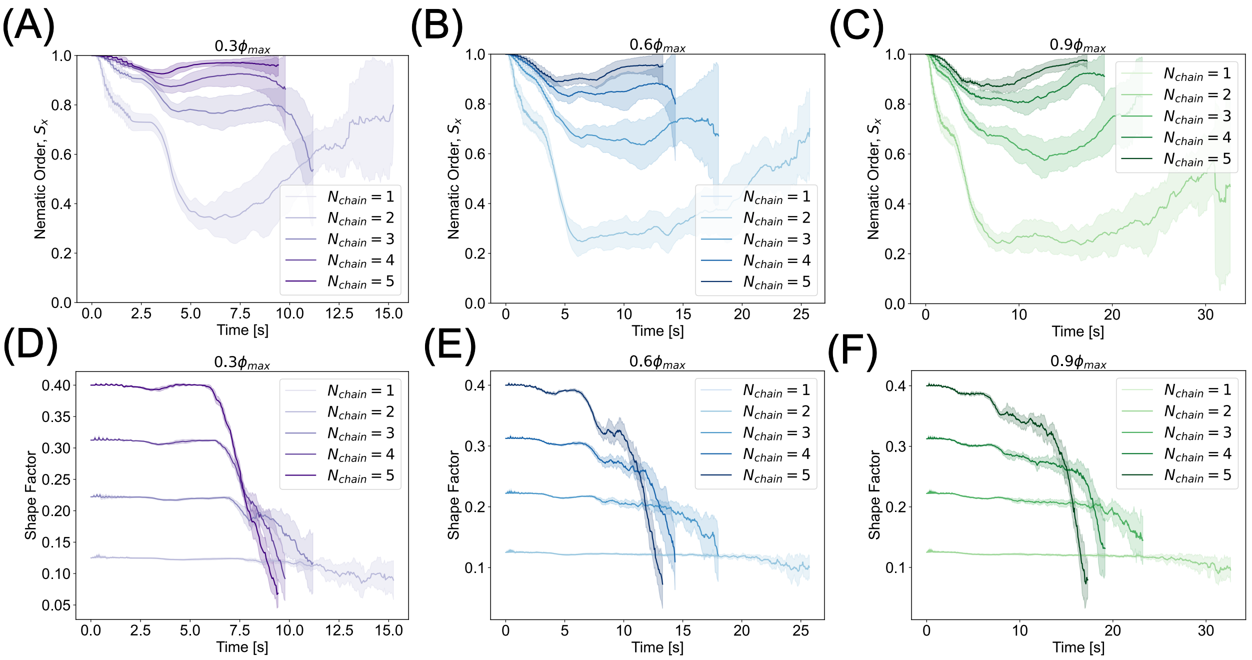

Figure S3: **Nematic and shape factor analysis of intermediate and sparse densities for varying mitochondrial aspect ratio.** (A-C) Nematic order time courses of increasing density for morphologies from granular ($N_{\text{chain}} = 1$) to elongated ($N_{\text{chain}} = 5$). Elongated mitochondria retain nematic order and recover unity while shorter morphologies show a reduced orientational order that does not recover. (D-F) Corresponding shape factors. Elongated mitochondria have a higher shape factor than shorter counterparts. Shape factor decays near the end of the trajectory as beads are exiting the simulation axon. Curves and shaded regions represent the mean and standard deviation, respectively, of ten simulations per parameter set.
